# Zebrafish parental progeny investment in response to cycling thermal stress and hypoxia: deposition of heat shock proteins but not cortisol

**DOI:** 10.1242/jeb.244715

**Published:** 2022-11-11

**Authors:** Michael Y.-T. Lim, Nicholas J. Bernier

**Affiliations:** Department of Integrative Biology, University of Guelph, 50 Stone Road East, Guelph, ON, Canada, N1G 2W1

**Keywords:** Non-genetic inheritance, Cellular stress response, Glucocorticoids, Environmental stressors, Diel cycling, Fish

## Abstract

The maternal match hypothesis predicts that maternal exposure to a stressor may help prepare offspring to cope with the same disturbance in later life. Although there is support for this hypothesis, the signals involved in non-genetic inheritance are unclear. In this study, we tested how adult zebrafish exposure to diel cycles of thermal stress (27–36°C), hypoxia (20–85% dissolved oxygen) or the combined treatment affects maternal and embryonic levels of cortisol and heat shock proteins (HSPs). While parental exposure to the thermal, hypoxic or combined treatment for 2 weeks did not affect whole-body cortisol levels, the combined exposure increased ovarian cortisol levels by 4-fold and reduced embryonic cortisol content by 60%. The combined treatment also elicited 3- and 19-fold increases in embryo transcripts involved in cortisol breakdown (*11bhsd2*) and export (*abcb4*), respectively. The thermal stress and combined exposure also elicited marked increases in ovary and embryo *hsp70a* (20- to 45-fold) and HSP70 (3- to 7-fold), and smaller increases in ovary and embryo *hsp90aa* and *hsp47* (2- to 4-fold) and in embryo HSP90 and HSP47 (2- to 6-fold). In contrast, except for increases in ovary *hsp90aa* (2-fold) and embryo HSP90 (3-fold), the hypoxia treatment had little effect on HSP expression and transfer. Overall, while the embryonic deposition of HSPs largely paralleled the ovarian cellular stress response, the inverse relationship between ovary and embryo cortisol levels suggests the existence of barriers against cortisol deposition in response to environmental stressors. We conclude that the endocrine and cellular stress responses make stressor-specific and distinct contributions to non-genetic inheritance.

## Teaser

**Summary:** The endocrine and cellular stress responses make stressor-specific and distinct contributions to non-genetic inheritance in zebrafish following chronic exposure to cycling thermal stress and/or hypoxia.

## INTRODUCTION

The maternal match hypothesis proposes that maternally experienced stress may help species cope with climate-induced environmental stressors. As described by Sheriff and Love (2013), the hypothesis predicts that offspring phenotypes will be better suited to respond to pressures found in the parental environment. Offspring phenotypes are modified by the transfer of maternal stress signals (social, resource-based or environmental; Sheriff and Love, 2013). Although such anticipatory maternal effects can result in seemingly maladaptive phenotypes when offspring are in mismatched conditions, performance and fitness may improve when offspring face similar stressful conditions to their mothers (DeWitt et al., 1998; Frazier and Roth, 2009; Kishimoto et al., 2017; Le Roy et al., 2017). Unlike older life stages, which can move in pursuit of more tolerable environmental conditions, embryos or larvae may be unable to immediately leave their nursery habitats and thus benefit from maternally induced phenotypic changes that improve offspring–environment compatibility (Figueira and Booth, 2010; Donelson et al., 2018). However, while several mechanisms may be involved in non-genetic inheritance, the specific biomolecules transferred from mothers to offspring in response to environmental stressors are unknown (Beldade et al., 2011; Gulyas and Powell, 2019).

Maternally derived glucocorticoids (GCs) are a potential candidate for shaping offspring phenotype in response to environmental stressors. As primary mediators of the vertebrate endocrine stress response, GCs increase in response to diverse stressors, and maternal GCs can be transferred to the next generation (Seckl and Meaney, 2004; Sheriff and Love, 2013; Sopinka et al., 2017a). In teleosts, maternal stress and experimentally elevated egg cortisol content (the primary GC in fish) can elicit various morphological, physiological and behavioural effects in offspring (McCormick, 1998; Eriksen et al., 2011; Capelle et al., 2016; Sopinka et al., 2016, 2017b; Best et al., 2017). Maternally derived cortisol also plays an important role in offspring development prior to *de novo* cortisol synthesis (Nesan and Vijayan, 2013). Moreover, several mechanisms can buffer deviations in egg cortisol content. For example, the enzymes 11β-hydroxysteroid dehydrogenase type 2 (11β-HSD2) and 20β-hydroxysteroid dehydrogenase type 2 (20β-HSD2), which catalyse the inactivation of cortisol to cortisone and 20β-hydroxycortisone (20β-HC), respectively, have high mRNA levels in the ovary and are expressed throughout embryonic development (Alderman and Vijayan, 2012; Tokarz et al., 2012; Faught et al., 2016). Newly fertilized eggs can also export cortisol via ATP binding cassette (ABC) transporters (e.g. ABCB4; Fischer et al., 2013; Paitz et al., 2016). Although maternal exposure to twice daily chasing for 2 weeks in coho salmon (*Oncorhynchus kisutch*; Stratholt et al., 1997) results in eggs with higher cortisol content, it is unclear in fish whether exposure to environmental stressors can increase egg cortisol content or produce phenotypes with improved performance under such conditions (Warriner et al., 2020).

Heat shock proteins (HSPs) may also be involved in communicating maternally derived stress. As key mediators of the cellular stress response, HSPs are involved in various cellular processes such as protein transport, folding and repair, and the regulation of signal transduction, and are important factors for environmental stress resistance (Sørensen et al., 2003; Chen et al., 2018). These molecular chaperones are grouped by size into HSP90, HSP70 and low molecular weight (16–47 kDa) HSP families (Basu et al., 2002). Members of these protein families are either constitutively expressed or inducible, and are regulated by heat shock factor 1 (HSF1; López-Olmeda and Sánchez-Vázquez, 2011). In teleosts, HSP90α (encoded as *hsp90aa*), HSP70 and HSP47 expression is inducible and upregulated by various stressors including heat (Krone and Sass, 1994; Delaney and Klesius, 2004; Todgham et al., 2005; Vinagre et al., 2012; Narum et al., 2013; Chadwick and McCormick, 2017; Levesque et al., 2019). Beyond their cytoprotective functions, HSPs play essential roles in developmental regulation (Queitsch et al., 2002; Krone et al., 2003; Yeyati et al., 2007; Takahashi et al., 2010). For example, maternal overexpression of low molecular weight HSPs increases embryo thermal tolerance in fruit flies (*Drosophila melanogaster*; Lockwood et al., 2017). In fish, although maternal HSP mRNAs may be transferred in the terminal stages of folliculogenesis (Santacruz et al., 1997; Knoll-Gellida et al., 2006), whether maternal exposure to environmental stressors affects this transfer remains to be established.

Elevated water temperatures and hypoxia, environmental stressors magnified by climate change and eutrophication, are significant threats to aquatic organisms (McBryan et al., 2013; Crozier and Hutchings, 2014; Jenny et al., 2016). For example, climate change-driven increases in global water temperatures, and local increases in temperature variability, are predicted to significantly reduce the spatial and temporal suitability of nursery habitats for fish spawning and larval rearing (Baumann et al., 2015; Tommasi et al., 2015; Booth et al., 2017). Likewise, global warming and eutrophication of aquatic habitats are linked to more severe, frequent and larger hypoxic zones (Díaz and Rosenberg, 2011; Jenny et al., 2016). Larger diel fluctuations in O_2_ availability and/or chronic exposure to hypoxic conditions in fish nurseries may also adversely affect spawning, offspring growth/development, and recruitment (Smith and Able, 2003; Cheek et al., 2009; Stierhoff et al., 2009; Campbell and Rice, 2014; Rooper et al., 2019; Mikloska et al., 2022). High temperatures and hypoxic conditions frequently co-occur in aquatic environments (McBryan et al., 2013). Warmer water temperatures not only increase aerobic metabolism and O_2_ demand of ectotherms but also increase the respiration rate of microorganisms that contribute to aquatic hypoxia, and reduce O_2_ solubility (Iriberri et al., 1985; Pörtner and Knust, 2007; Schulte, 2015). Despite these environmental stressors posing an increasing threat to the well being and survival of fish, few studies have examined the potential mechanisms involved in mediating the transgenerational effects of climate change (Donelson et al., 2018; Ryu et al., 2018). To our knowledge, whether biomolecules such as GCs or HSPs are transferred between mother and offspring in response to heat stress and/or hypoxia is not known.

Therefore, the objective of this study was to determine whether adult zebrafish exposure to heat stress and/or hypoxia is associated with the maternal transfer of GCs and HSPs to offspring. We selected zebrafish for this study as they experience variable temperatures (∼6–38°C) and O_2_ levels (∼1–350% dissolved O_2_) in their natural habitat (Khan et al., 1970; Engeszer et al., 2007; Spence et al., 2008). Consistent with the hypothesis that mothers transfer stress-related signals to produce offspring that are suited to respond to similar selective pressures to those found in the parental environment, we predicted that adult fish chronically exposed to heat stress and/or hypoxia will share information about their environmental conditions by increasing the transfer of signals from the endocrine and/or cellular stress responses to their offspring. Specifically, we exposed adult zebrafish for 2 weeks to diel cycles of elevated temperatures and/or hypoxia, allowed the fish to spawn, and quantified components of the endocrine (cortisol, cortisone and 20β-HC levels; *11bhsd2*, *20bhsd2* and *abcb4* mRNA levels) and cellular (*hsf1*, *hsp70a*, *hsp90aa* and *hsp47* mRNA levels; HSP70, HSP90 and HSP47 protein levels) stress response in adult female tissues and in their embryos prior to zygotic transcription.

## MATERIALS AND METHODS

### Experimental animals

Adult zebrafish, *Danio rerio* (F. Hamilton 1822), were acquired from AQuality Tropical Fish Wholesale (Mississauga, ON, Canada). The *F*_0_ generation was reared in a recirculating multi-tank system (ZebTEC rack, Tecniplast USA, West Chester, PA, USA) for at least 3 months after acquisition, and maintained on a 12 h:12 h light:dark cycle at 27.5°C under normoxic conditions (>85% dissolved oxygen, DO) and pH ∼7.2. Adult zebrafish were held in 3.5 l tanks at a density of ∼25 fish per tank and fed twice daily to satiation with 0.5 mm sinking pellets (Northfin, Toronto, ON, Canada) and once daily with brine shrimp (Hikari USA, Hayward, CA, USA). All experiments were performed in accordance with guidelines set by the Canadian Council for Animal Care and were approved by the University of Guelph's Animal Care Committee.

A total of 216 adult *F*_0_ fish per treatment group were separated based on secondary sexual characteristics into 32, ∼1.4 l custom-made mesh-bottom spawning baskets, at a density of 6 females (24 baskets) or 9 males (8 baskets) per basket. Baskets were separated and submerged into four, ∼180 l aquaria with ∼120 l of water. Each aquarium contained 6 female and 2 male baskets arranged randomly in two rows and four columns. Tanks were held at a base temperature of 27°C, supplied with deionized City of Guelph tap water (pH ∼7.1) combined with a sea salt mixture (0.5 g l^−1^; Instant Ocean, Spectrum Brands Inc., Blacksburg, VA, USA), aerated (>85% DO; >6.77 mg O_2_ l^−1^), and maintained on a 12 h:12 h light:dark cycle. Water temperature and % DO were maintained using an automated monitoring and control system (Argus Control Systems Ltd, Surrey, BC, Canada). Temperature and DO readings were periodically verified with a hand-held meter (Handy Polaris, Oxyguard, Farum, Denmark) throughout the experiment. All tanks were equipped with a ∼113 l sponge filter (Hydra Aquatics, Dania Beach, FL, USA). Daily ∼20% water changes were carried out, and water quality tests (API Freshwater Water Master Test Kit, API, Chalfont, PA, USA) were performed twice weekly to verify nitrogenous waste levels remained low.

### Experimental design

**Fig. 1.**
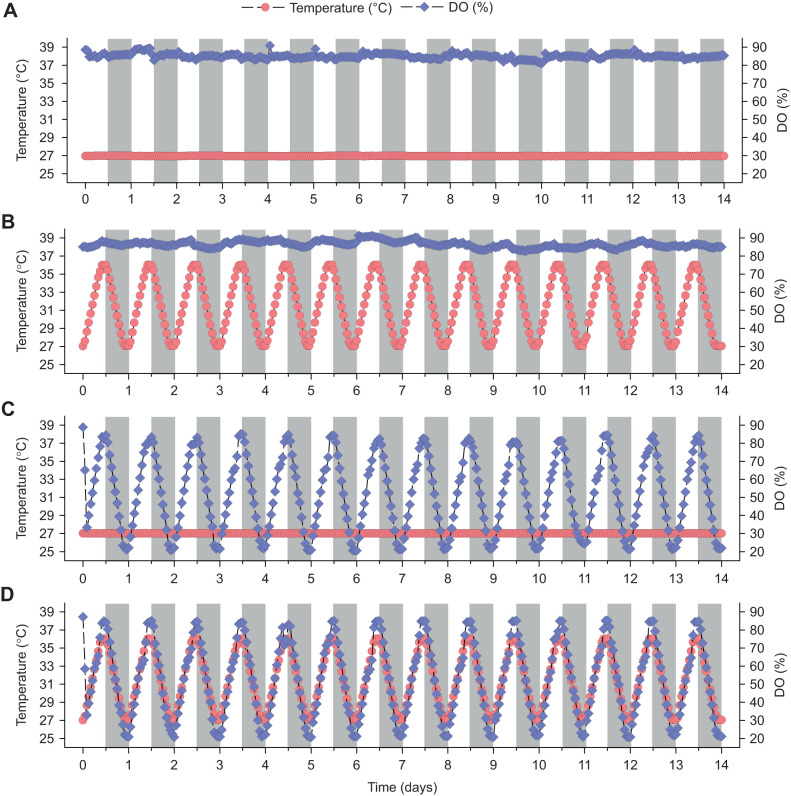
**Experimental treatments.** Temperature and percentage dissolved oxygen (DO) profiles of the (A) control, (B) cycling temperature, (C) cycling hypoxia and (D) combined exposure treatments. In B–D, temperature and/or DO was increased during the light phase (white background) and decreased during the dark phase (grey background). Note that maximum temperature and minimum % DO levels are offset from each other by 12 h. Although temperature and DO measurements were taken every second, only the 60 min running means are shown over the course of 14 days for presentation clarity.

Fish were initially held under control conditions (27°C, ∼85% DO) to acclimate for 3 days, and then exposed to one of four treatments for 14 days: (1) control conditions (Fig. 1A; mean±s.d., 26.94±0.02°C and 85.03±1.60% DO), (2) cycling temperature (Fig. 1B), (3) cycling hypoxia (Fig. 1C) and (4) cycling temperature and hypoxia (combined exposure; Fig. 1D). A treatment period of 14 days was chosen as egg development in zebrafish is asynchronous, taking place within the ovary over the course of 5–10 days (Clelland and Peng, 2009; Hisaoka and Firlit, 1962). Fish were fed thrice daily with floating flake food (TetraMin Tropical Flakes, Blacksburg, VA, USA) throughout the acclimation and treatment periods. To control for possible variation in parental exposure conditions (e.g. light intensity, proximity to airstones), spawning baskets in the ∼180 l aquaria were rotated without emersion one position clockwise every day during the acclimation and treatment periods. In the cycling temperature treatment, adult fish were maintained under normoxia (>85% DO) and exposed to a daily temperature cycling regime ranging between 27 and 36°C. Warming began with light phase onset, and the temperature was held constant until cooling began with the onset of the dark phase. Warming/cooling rates were ∼1°C h^−1^. The average temperature and DO were 31.49±3.18°C and 86.47±1.74%, respectively (Fig. 1B). Fish in the cycling hypoxia treatment were maintained at 27°C and exposed to a daily hypoxic cycling regime between 20% and 85% DO. Using a mixture of compressed air and N_2_ gas, the % DO was gradually lowered until it reached 20% (1.58 mg l^−1^) during the dark phase and it was raised to 85% (6.72 mg l^−1^) during the light phase. The rate of change of DO was ∼7.2% h^−1^. The average temperature and DO were 27.01±0.02°C and 52.41±21.18%, respectively (Fig. 1C). In the combined exposure treatment, fish were exposed to a combined cycling temperature and hypoxia regime which peaked at 36°C and 85% DO during the light phase and reached minimum levels of 27°C and 20% DO during the dark phase (Fig. 1D). The average temperature and DO in the combined exposure treatment were 31.50±3.17°C and 52.22±21.26%, respectively (Fig. 1D). In all treatments, during each diel cycle, temperature and DO conditions were kept constant for a period of 3 h after 9 h of gradual increase or decrease. Because of the physical properties of oxygen solubility in water, % DO changes were scaled to holding temperature and not to 27°C conditions.

On the evening of the 13th day of the treatment period, 3 males from each treatment group were added to each female basket for a total of 6 mixed-sex baskets per treatment. On the morning of the 14th day, eggs were collected within ∼1 h after light onset (08:00 h) via a container placed under the mesh-bottom spawning baskets. Embryos were transferred into Petri dishes (15×100 mm) with egg water (60 µg ml^−1^ Instant Ocean; 0.0004% Methylene Blue mixed with de-ionized water) for counting. Both dead and live embryos were counted, and dead embryos were removed. The average fecundity and percentage viability (percentage of live embryos/total number of embryos) was determined per female per basket for each treatment group to quantify measures of spawning success. Live 1 h post-fertilization (1 hpf) embryos were then snap frozen on dry ice prior to storage at −80°C for subsequent analyses. Following embryo collection, adult zebrafish were euthanized (09:00 h) by rapid cooling in ice water (<4°C) with MS-222 (0.05%; Syndel International, Qualicum Beach, BC, Canada) and blotted dry to measure body mass (BM) and fork length (FL), and to quantify Fulton's condition factor as *K*=(BM FL^−3^). The ovaries, gills and bodies of females were separated, and then snap frozen on dry ice prior to storage at −80°C for subsequent analyses. To assess whether the 14 day cycling temperature, cycling hypoxia and combined exposure treatments elicit endocrine and cellular stress responses in females, and to determine whether maternal signals of stress are deposited into the eggs, we quantified glucocorticoids (adult female bodies: cortisol, cortisone, 20β-HC; ovaries and embryos: cortisol), mRNA levels (female gills*: 11bhsd2*, *20bhsd2*, *hsf1*, *hsp70a*, *hsp90aa* and *hsp47*; ovaries and embryos: *11bhsd2*, *20bhsd2*, *abcb4*, *hsf1*, *hsp70a*, *hsp90aa* and *hsp47*) and protein levels (female gills, ovaries and embryos: HSP70, HSP90 and HSP47). Although liver has been used to quantify zebrafish HSP induction (e.g. Rabergh et al., 2000), preliminary analysis in this study observed extremely variable mRNA levels between liver samples from the same treatment group (data not shown). Therefore, we opted to sample gills (i.e. gill arches) for mRNA and protein analysis of HSP induction, as has also been done in previous studies (Rabergh et al., 2000; Basu et al., 2002). Ovary mRNA and protein were extracted from ∼50 mg sections, and embryo mRNA and protein were extracted from pools of 30 embryos (sampled before zygotic transcription; Kane and Kimmel, 1993).

### Steroid extraction

Adult ‘whole-body’ samples were composed of female bodies excluding the ovaries and gills. Bodies were homogenized (Polytron homogenizer, Brinkmann Instruments, Rexdale, ON, Canada) on ice in glass culture tubes (16×100 mm) with 4 ml of homogenizing buffer (Fuzzen et al., 2010), spiked with 10 µl of cortisol-d_4_ (2.5 µg ml^−1^ cortisol-9,11,12,12-d_4_; D-5280, C/D/N Isotopes Inc., Pointe-Claire, QC, Canada; dissolved in 1:1 methanol and water) to quantify extraction recovery, and sonicated (Sonics and Materials Incorporated, Danbury, CT, USA). Homogenates were then extracted twice with methanol and further purified using C_18_ solid phase extraction columns [300 mg octadecyl (C_18_), 3 ml column; Cleanert S C18-N SPE, Agela Technologies, Tianjin, China] as per Fuzzen et al. (2010). Samples recovered from the C_18_ columns were dried with compressed air at room temperature in a fume hood, then reconstituted in 300 µl of methanol, transferred to 350 µl glass vials and dried without air flow at room temperature in a fume hood overnight. Samples were then reconstituted in 90 µl of 1:1 methanol and water, and spiked with an additional 10 µl of 17β-estradiol 3-benzoate (200 ng ml^−1^; dissolved in 1:1 methanol and water; Steraloids Inc., Newport, RI, USA) to quantify recovery of steroids through mass spectrometry analysis.

Ovaries and embryos were processed similar to bodies, but with reduced volumes during the methanol extraction step (i.e. 500 µl homogenizing buffer, 2 ml methanol per wash), and reduced columns and volumes during the C_18_ column purification step [100 mg octadecyl (C_18_), 1 ml column, Agela Technologies; 1 ml per reagent used in C18 column purification]. After samples recovered from the C_18_ columns were dried, they were reconstituted in 110 µl of diluted extraction buffer (as per the manufacturer's instructions; Neogen, Lexington, KY, USA).

### Steroid quantification

Adult whole-body cortisol, cortisone and 20β-hydroxycortisone were measured via liquid chromatography (LC) tandem mass spectrophotometry (MS/MS). LC-MS/MS was performed on an Agilent 1200 Series binary pump (Agilent Technologies Inc., Santa Clara, CA, USA) coupled to a QTRAP 5500 triple-quadrupole mass spectrometer (SCIEX, Concord, ON, Canada). Reverse phase HPLC was performed using a Kinetex C18 column (100×2.1 mm, 2.6 µm particle size; cat. no. 686 551-8, Phenomenex, Torrance, CA, USA). The mobile phases consisted of 0.01% acetic acid in water (A) and 0.01% acetic acid in methanol (B). The method was optimized for peak shape for the different analytes measured. A gradient program was used for the HPLC separation at a flow rate of 0.3 ml min^−1^. The initial solvent composition was 0:100 (A:B) for 9 min, then ramped to 100:0 (A:B) over 6 min. Conditions were held for 2 min before returning to initial conditions for the next injection. Prior to MS analysis, retention times, mass transition parameters and limits of detection were determined by infusion of pure standards (Table S1; Steraloids Inc.). MS analysis was performed in positive electrospray ionization mode with multiple reaction monitoring (MRM) to select both parent and characteristic daughter ions specific to each analyte simultaneously from a single injection. Nitrogen was used as the nebulizing, turbo spray and curtain gas. Curtain gas, collisional activated dissociation (CAD), gas temperature (TEM), ion spray voltage (IS), source gas 1 (GS1), source gas 2 (GS2) and entrance potential (EP) parameters were set to 35, medium, 500°C, 4500 V, 40, 30 and 10, respectively. Each target was then uniquely identified by the parent-to-daughter ion mass transition and the specific retention time (see Table S1). Data were collected and analysed by Analyst v1.6.2 (SCIEX). Quantitative analysis was based on calibration curves for each analyte (spiked with the same amount of internal standard (cortisol-d_4_) and loading standard (17β-estradiol 3-benzoate). To normalize sample concentrations of cortisol, cortisone and 20β-hydroxycortisone, first, internal and loading standard concentrations were calculated. When calculating sample concentrations, known differences in internal and loading standard concentrations (2.5 µg ml^−1^ and 200 ng ml^−1^, respectively) were corrected for. LC-MS/MS analysis was performed at the Analytical Facility for Bioactive Molecules of the Hospital for Sick Children, Toronto, ON, Canada.

Glucocorticoid levels in the ovary and embryo samples fell below the LC MS/MS limits of detection (Table S1). Therefore, ovary and embryo cortisol levels were quantified using a commercial ELISA kit (Neogen, Lexington, KY, USA), and neither ovary nor embryo cortisone and 20β-hydroxycortisone levels were quantified in this study. All standards and samples were run in duplicate. The lower detection limit of the assay was 10 pg ml^−1^. The intra- and inter-assay coefficients of variation were 9.3% (*n*=4) and 9.5% (*n*=3), respectively, and a serial dilution of pooled embryo extract gave a displacement curve that was parallel to the standard curve. Cortisol values were corrected for extraction efficiency (85%). According to the manufacturer, the cross-reactivity of the commercial antibody to other steroids is as follows: prednisolone 47.4%, cortisone 15.7%, 11-deoxycortisol 15.0%, prednisone 7.83%, corticosterone 4.81%, 6β-hydroxycortisol 1.37%, 17-hydroxyprogesterone 1.36%, deoxycorticosterone 0.94%. Steroids with cross-reactivity ≤0.06% are not presented.

### RNA extraction, cDNA synthesis and mRNA quantification

Quantification of mRNA levels was completed via quantitative real-time PCR (qPCR) as per Williams and Bernier (2020). Briefly, tissues of interest were homogenized in 0.5 ml of Ribozol RNA extraction reagent (Thermo Fisher Scientific, Waltham, MA, USA) using a bead beater (Precellys Evolution, Bertin Technologies, Montigny-le-Bretonneux, France; 5500 rpm, 3×30 s separated by 5 s pauses). To increase recovery, 10 µg of RNA-grade glycogen (Thermo Fisher Scientific) was added to each sample before precipitation in isopropanol at −80°C overnight. Total RNA was quantified via a Nanodrop spectrophotometer (Nanodrop 2000 UV-vis, Thermo Fisher Scientific). From each sample, 100 ng was treated with DNase (Quanta Biosciences, Beverly, MA, USA) and used to synthesize cDNA using Quanta qScript (Quanta Biosciences) as per the manufacturer's instructions. Separate samples were treated identically without the addition of reverse transcriptase (RT) or without the presence of RNA to verify the absence of genomic DNA or contaminated reagents.

qPCR was performed on a CFX96 system (Bio-Rad, Hercules, CA, USA) using 20 µl reaction volumes that contained 10 µl of master mix (SsoAdvanced Universal SYBR Green Supermix, Bio-Rad), 5 µl of 10-fold diluted first-strand cDNA template or no-RT controls, and 2.5 µl of both forward and reverse primers (0.4 µmol l^−1^; Table S2). Default cycling conditions were used and followed by a melting curve analysis to verify the specificity of each PCR product. Samples were analysed in triplicate and verified to have unimodal dissociation curves that matched the predicted melting point temperatures. To account for differences in amplification efficiency, standard curves were constructed for each gene using known dilutions of cDNA from gill, ovary or embryo samples. Input values for each gene were obtained by fitting average cycle threshold (Ct) values to the antilog of the gene-specific standard curves, and thereby correcting for differences in amplification efficiency. To correct for any template input and/or transcriptional efficiency differences, input values were normalized to the geometric mean of the housekeeping genes elongation factor 1α (*ef1α*) and ribosomal protein L13A (*rpl13a*). Gene expression data are reported as fold-change relative to the control treatment mean value.

### Protein quantification

Soluble protein was extracted from female adult tissues (gills, ovaries) and embryos. Briefly, samples (2 gill arches, ∼50 mg ovary, 30 embryos) were homogenized with radio immune-precipitation assay lysis buffer (200 µl for gills, 300 µl for ovaries and embryos) with protease inhibitors (0.574 mmol l^−1^ PMSF, 2 mmol l^−1^ EDTA) using a bead beater (Precellys Evolution; 6000 rpm, 3×15 s separated by 15 s pauses). Samples were then mixed on an orbital shaker (30 min; 4°C), centrifuged at 11,700 ***g*** for 20 min (4°C), and protein concentration in the supernatant was determined with a Bradford assay (Bio-Rad Protein Assay Dye Reagent, Bio-Rad). Samples were diluted to 1.98 µg µl^−1^ to a total aliquot volume of 45 µl. Each aliquot was combined with 15 µl of 4× Laemmli buffer to a final concentration of 1.49 µg µl^−1^, vortexed, then incubated at 65°C for 10 min before being pulse spun and stored at −20°C.

Gel electrophoresis was performed on diluted samples alongside a protein ladder (PageRuler prestained protein ladder, Thermo Scientific), standards (diluted to 10 ng µl^−1^), and a blank (7.5 µl of ddH_2_O and 2.5 µl of 4× Laemmli buffer). The standards consisted of rat recombinant HSP70/HSP72 (cat. no. ADISPP7580, Enzo Life Sciences, Farmingdale, NY, USA) and native human HSP90 (cat. no. ADISPP770D, Enzo Life Sciences). A commercial HSP47 standard was not available, so a positive control made from a pool of gill tissues from heat-stressed zebrafish was used instead and verified with a 1:2000 synthetic HSP47/SERPINH1 blocking peptide (cat. no. 33R-2012, Fitzgerald Industries International, Acton, MA, USA). Samples (20 μg protein) were separated on an 8% SDS-polyacrylamide gel (with 5% stacking gel) and transferred to a polyvinylidene difluoride (PVDF) membrane (Immobilon-P, Merck Millipore Ltd, Carrigtwohill, Country Cork, Ireland). Membranes were then blocked for 1 h at 20°C in 5% non-fat milk powder dissolved in Tris-buffered saline with Tween 20 (TBST). Incubations with primary antibody against HSP47 (1:1000; polyclonal rabbit HSP47/SERPINH1, cat. no. 20R-1310, Fitzgerald Industries International), HSP70 (1:5000; polyclonal rabbit HSP70/HSC70, cat. no. AS05083A, Agrisera, Vännäs, Sweden) and HSP90 (1:2500; monoclonal mouse HSP90, cat. no. SMC-107, StressMarq Biosciences Inc., Victoria, BC, Canada) were carried out overnight at 4°C. According to the manufacturers, the HSP70 antibody recognizes both the inducible (HSP70) and constitutive (HSC70) isoforms, and the HSP90 antibody primarily recognizes the beta isoform (HSP90β) but may also detect the alpha isoform (HSP90α). Secondary antibody incubations for HSP47/HSP70 (1:20,000; polyclonal goat anti-rabbit, cat. no. AS09602, Agrisera) and HSP90 (1:5000 polyclonal goat anti-mouse, cat. no. ab5870, Abcam, Cambridge, UK) were performed for 1 h at 20°C. All antibodies were diluted in 1% non-fat milk powder dissolved in TBST. Chemiluminescent detection of protein bands was performed using Superbright ECL (cat. no. AS16ECL-S, Agrisera). Blots were imaged using a Bio-Rad ChemiDoc MP Imaging System (Universal Hood III, Bio-Rad) and analysed with ImageJ (National Institutes of Health, Bethesda, MD, USA). Note that equal proteins were loaded on each gel and verified visually via Coomassie staining of PVDF membranes after immunodetection (Welinder and Ekblad, 2011). As specific concentrations were not of interest, all band densities were expressed relative to control tissues for each protein.

### Statistical analysis

All data are presented as means±s.e.m unless otherwise stated. Differences between treatments were analysed by one-way ANOVA and followed by a Holm–Šidák *post hoc* test when the ANOVA was significant. Square-root or log_10_ transformation was applied if a Shapiro–Wilk test for normality or Bartlett test for homogeneity of variances among groups was significant. If transformation proved insufficient to meet the ANOVA assumptions, the Kruskal–Wallis one-way ANOVA on ranks was used followed by Tukey *post hoc* tests, or a Dunn's *post hoc* test if *n* values were unequal. Any outliers that were determined to be greater than or less than the 1.5× inter-quartile range from the upper quartile or lower quartile, respectively, were removed from the gene expression dataset. No more than two outliers were found in any one treatment group and were attributed to low RNA integrity. All tests were conducted in SigmaPlot 12.5 (SysStat Software, San Jose, CA, USA) and α was set at 0.05.

## RESULTS

### Effects of cycling temperature and/or hypoxia on adult female body condition and spawning success

Relative to the control treatment, exposure for 14 days to cycling temperature, hypoxia or the combined exposure treatment did not affect adult female body condition (Table 1; *H*=2.72, d.f.=3, *P*=0.437). Similarly, fecundity in the treatments with cycling temperature and/or hypoxia did not differ from the control treatment. However, adult females exposed to cycling hypoxia for 14 days had fewer embryos than females exposed to the cycling temperature treatment (*H*=9.15, d.f.=3, *P*=0.027). In contrast, although embryo viability did not differ between the three cycling treatments and the control group, embryos from parents exposed to cycling temperature had a lower viability than embryos from either the cycling hypoxia or the combined exposure treatment (*H*=12.93, d.f.=3, *P*=0.005).

**Table 1.**
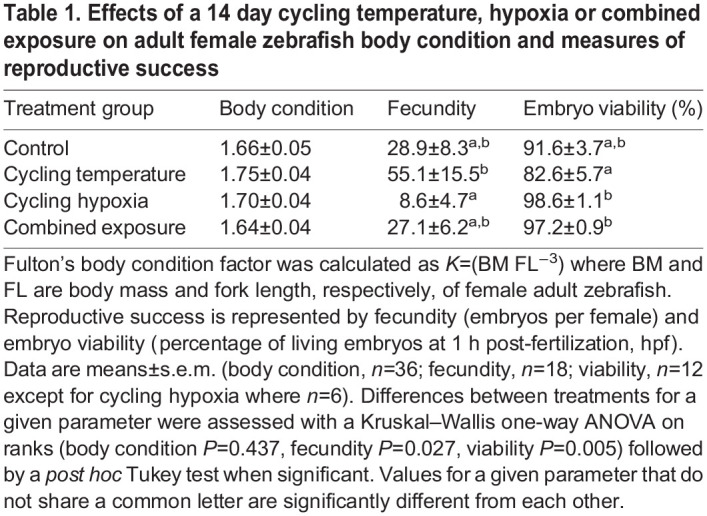
**Effects of a 14** **day cycling temperature, hypoxia or combined exposure on adult female zebrafish body condition and measures of reproductive success**

### Effects of cycling temperature and/or hypoxia on the GC stress response in adult females and their embryos

**Fig. 2.**
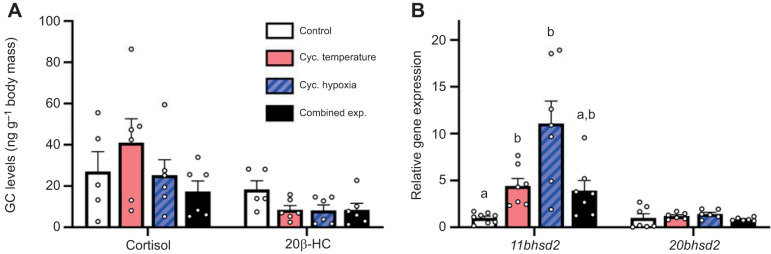
**Effects of experimental treatments on whole-body glucocorticoid (GC) levels and gill gene expression in adult female zebrafish.** (A) Whole-body cortisol and 20β-hydroxycortisone (20β-HC) levels. (B) Gill *11bhsd2* and *20bhsd2* relative gene expression. Gene expression values were normalized to the geometric mean of *ef1α* and *rpl13a* expression, and the expression ratio for each gene is presented relative to the control treatment. Values are means+s.e.m. (cortisol and 20β-HC, *n*=5–6; *11bhsd2*, *n*=7–8; *20bhsd2*, *n*=6–7). Cortisol and 20β-HC levels were compared with one-way ANOVA (*P*=0.298 and *P*=0.091, respectively). Statistical differences between gene expression values were determined by Kruskal–Wallis one-way ANOVA followed by *post hoc* Dunn's test (*11bhsd2*, *P*<0.001; *20bhsd2*, *P*=0.267). Values for a given parameter that do not share a common letter are significantly different from one another.

Whole-body cortisol (*F*=1.32, d.f.=3, *P*=0.298) and 20β-HC (*F*=2.50, d.f.=3, *P*=0.091) levels did not differ between treatments (Fig. 2A) and cortisone levels were below the limit of detection in all samples, suggesting that the treatments did not elicit a sustained endocrine stress response. However, we observed a significant difference in gill *11bhsd2* expression between treatments (Fig. 2B; *H*=18.66, d.f.=3, *P*<0.001). Relative to the control treatment, the cycling temperature, hypoxia and combined exposure treatments increased *11bhsd2* mRNA levels ∼4.4-, ∼11.1- and ∼3.9-fold, respectively, but the change did not reach statistical significance in the combined exposure treatment. In contrast, gill *20bhsd2* expression did not differ between treatments (*H*=3.87, d.f.=3, *P*=0.276).

**Fig. 3.**
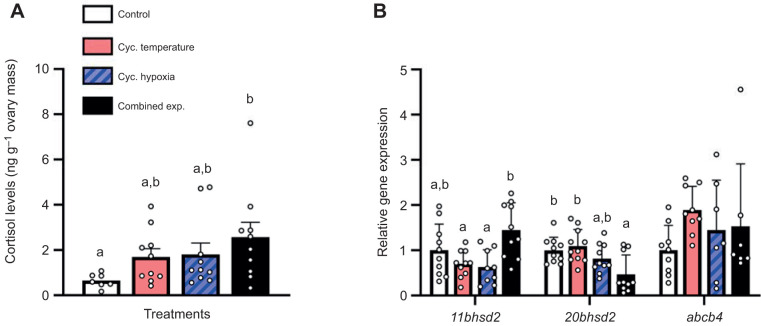
**Effects of experimental treatments on ovary cortisol levels and gene expression in adult female zebrafish.** (A) Ovary cortisol levels. (B) Ovary *11bhsd2*, *20bhsd2* and *abcb4* relative gene expression. Gene expression data were normalized and expressed as stated in Fig. 2. Values are means+s.e.m. (cortisol, *n*=10 except for control where *n*=7; *11bhsd2*, *n*=9–10; *20bhsd2*, *n*=10–11; *abcb4*, *n*=7–9). Cortisol levels were square-root transformed prior to being compared with a one-way ANOVA with a *post hoc* Holm–Šidák test (*F*=2.906, d.f.=3, *P*=0.049). Statistical differences between gene expression values were determined by a one-way ANOVA with a *post hoc* Holm–Šidák test (*11bhsd2*, *P*=0.003) or a Kruskal–Wallis one-way ANOVA on ranks with *post hoc* Dunn's test (*20bhsd2*, *P*=0.010; *abcb4*, *P*=0.091). Values for a given parameter that do not share a common letter are significantly different from one another.

The cycling temperature, hypoxia and combined exposure treatments increased ovarian cortisol levels ∼2.6-, ∼2.8- and ∼3.9-fold from control treatment levels, respectively; however, the response was variable and only reached statistical significance in the combined exposure treatment (Fig. 3A; *F*=2.91, d.f.=3, *P*=0.049). While ovarian *11bhsd2* expression did not differ between the control and the three cycling treatments, it was increased ∼2.2-fold in the combined exposure treatment versus either the cycling temperature or cycling hypoxia treatment (Fig. 3B; *F*=5.75, d.f.=3, *P*=0.003). In contrast, ovarian *20bhsd2* expression was lower in the combined exposure treatment compared with both the control and cycling temperature treatments (*H*=11.41, d.f.=3, *P*=0.010). Moreover, ovarian *abcb4* expression did not differ between treatments (*H*=6.47, d.f.=3, *P*=0.091).

**Fig. 4.**
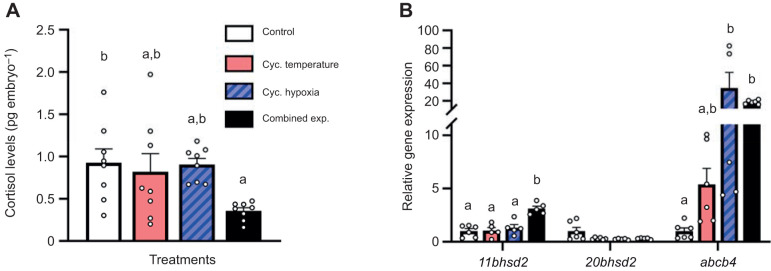
**Effects of parental treatment on zebrafish embryo cortisol levels and gene expression.** (A) Cortisol levels and (B) *11bhsd2*, *20bhsd2* and *abcb4* relative gene expression of ∼1 h post-fertilization (hpf) embryos derived from adult zebrafish exposed to the experimental treatments. Gene expression data were normalized and expressed as stated in Fig. 2. Values are means+s.e.m. (cortisol, *n*=8; *11bhsd2*, *20bhsd2* and *abcb4*, *n*=5–6). Cortisol levels were compared with a one-way ANOVA with a *post hoc* Holm–Šidák test (*F*=3.631, d.f.=3, *P*=0.025). Statistical differences between gene expression values were determined by a one-way ANOVA with a *post hoc* Holm–Šidák test (*11bhsd2*, *P*<0.001) or a Kruskal–Wallis one-way ANOVA on ranks with a *post hoc* Dunn's test (*20bhsd2*, *P*=0.114; *abcb4*, *P*=0.001). Values for a given parameter that do not share a common letter are significantly different from one another.

Interestingly, while embryo cortisol levels did not differ between the control, cycling temperature and cycling hypoxia treatments, embryos from parents in the combined exposure treatment had cortisol levels that were ∼60% lower than those of control embryos (Fig. 4A; *F*=3.63, d.f.=3, *P*=0.025). Embryonic *11bhsd2* mRNA levels also did not differ between the control, cycling temperature and cycling hypoxia treatments, but were increased 3.1-fold in the combined exposure treatment versus control embryos (Fig. 4B; *F*=15.06, d.f.=3, *P*<0.001). In contrast, embryonic *20bhsd2* expression did not differ between treatments (*H*=5.94, d.f.=3, *P*=0.114). Finally, relative to the control treatment, embryonic *abcb4* mRNA levels increased 5.4-, 34.5- and 18.5-fold in the cycling temperature, cycling hypoxia and combined exposure treatments, respectively, with changes that reached statistical significance in the cycling hypoxia and combined exposure treatments (*H*=16.07, d.f.=3, *P*=0.001).

### Effects of cycling temperature and/or hypoxia on the cellular stress response in adult females and their embryos

**Fig. 5.**
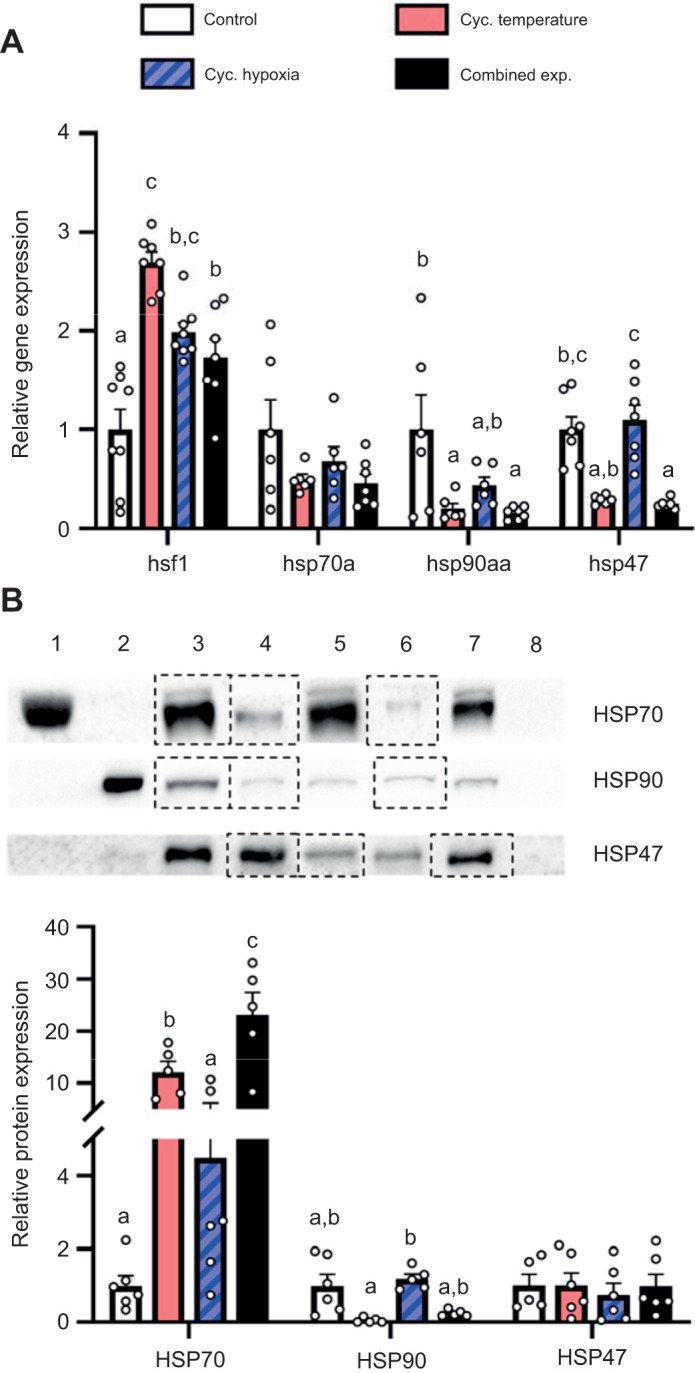
**Effects of experimental treatments on the gill cellular stress response in adult female zebrafish.** (A) Gill *hsf1*, *hsp70a*, *hsp90aa* and *hsp47* relative gene expression. (B) Representative western blot and HSP70, HSP90 and HSP47 relative protein expression. Gene expression data were normalized and expressed as stated in Fig. 2. Western blot shows HSP70 standard (lane 1), HSP90 standard (lane 2), pool of heat-stressed gills (positive control; lane 3), control treatment (lane 4), cycling temperature treatment (lane 5), cycling hypoxia treatment (lane 6), combined exposure treatment (lane 7) and blank (lane 8). Dashed lines around a lane represent the splicing of separate gel images. Protein expression was normalized to Coomassie stain band intensity and expressed relative to the control treatment for each protein. Values are means+s.e.m. (*hsf1*, *n*=7–8; *hsp70a*, *hsp90aa* and *hsp47*, *n*=6–7; HSP70, HSP90 and HSP47, *n*=5–6). Statistical differences between gene expression values were determined by Kruskal–Wallis one-way ANOVA followed by *post hoc* Dunn's test (*hsf1*, *P*<0.001; *hsp70a*, *P*=0.363; *hsp90aa*, *P*<0.001; *hsp47*, *P*<0.001). HSP70 protein expression was square-root transformed prior to analysis; statistical differences between protein expression values were determined by one-way ANOVA followed by *post hoc* Holm–Šidák tests (HSP70, *P*<0.001; HSP90, *P*=0.027) or a Kruskal–Wallis one-way ANOVA (HSP47, *P*=0.868). Values for a given parameter that do not share a common letter are significantly different from one another.

Relative to control conditions, the cycling temperature, hypoxia and combined exposure treatment increased gill *hsf1* mRNA levels in the adult females ∼2.7-, ∼2.0- and ∼1.7-fold, respectively (Fig. 5A; *H*=22.05, d.f.=3, *P*<0.001). However, gill *hsp70a* expression did not differ between treatments (*H*=3.20, d.f.*=*3, *P*=0.363). Gill *hsp90aa* expression in the cycling temperature and combined exposure treatments decreased by ∼80% relative to the control treatment (*F*=5.66, d.f.=3, *P*=0.005). Similarly, gill *hsp47* expression in the combined exposure treatment was ∼80% lower than in the control and cycling hypoxia treatments, and ∼70% lower in the cycling temperature treatment than in the cycling hypoxia treatment (*H*=19.19, d.f.=3, *P*<0.001). In contrast to the gene expression results, gill HSP70 protein levels increased in the cycling temperature and combined exposure treatment relative to the control (∼12.1- and ∼23.1-fold, respectively) and cycling hypoxia (∼2.7- and ∼5.1-fold, respectively) treatments (Fig. 5B; *F*=22.18, d.f.=3, *P*<0.001). Gill HSP90 protein levels in the cycling regimes did not differ from the control, but were ∼96% lower in the cycling temperature treatment than in the cycling hypoxia treatment (*F*=4.52, d.f.=3, *P*=0.027). Lastly, gill HSP47 levels did not differ across treatments (*H*=0.72, d.f.=3, *P*=0.868).

**Fig. 6.**
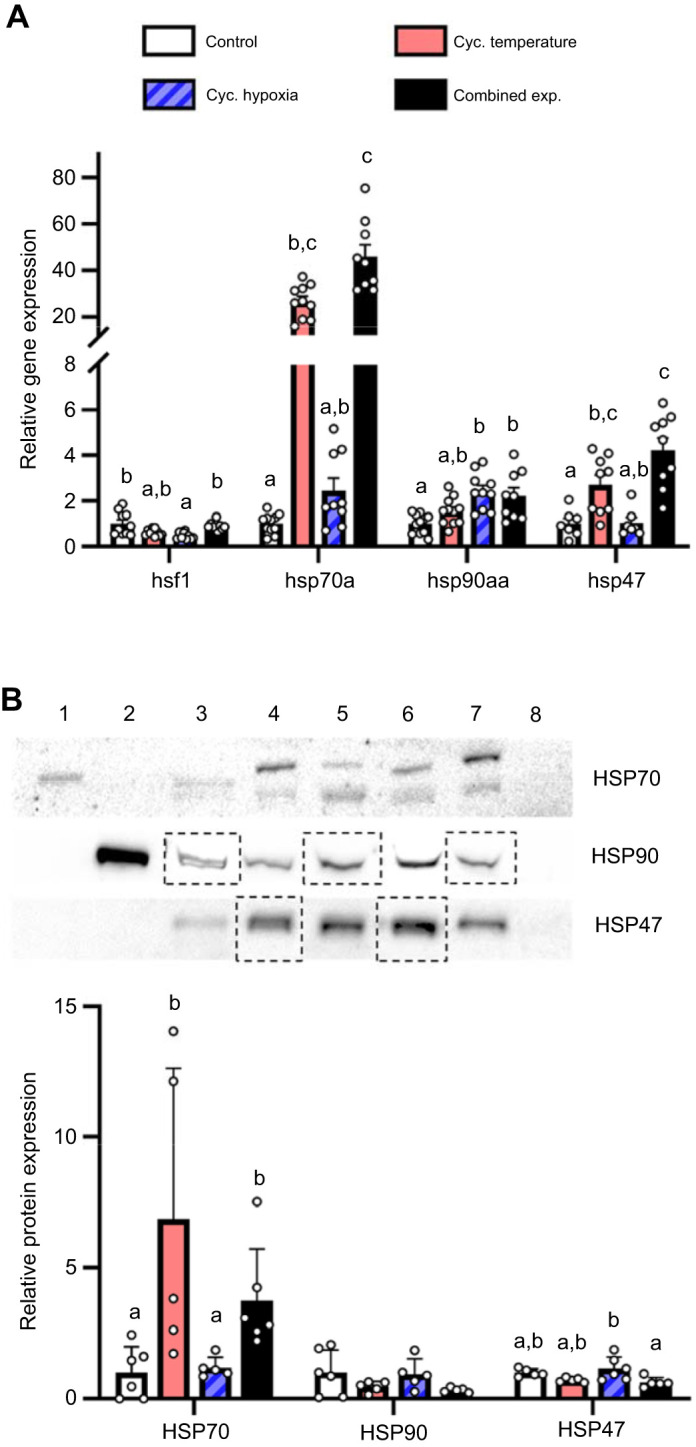
**Effects of experimental treatments on the ovary cellular stress response in adult female zebrafish.** (A) Ovary *hsf1*, *hsp70a*, *hsp90aa* and *hsp47* relative gene expression. (B) Representative western blot and HSP70, HSP90 and HSP47 relative protein expression. Gene expression data were normalized and expressed as stated in Fig. 2. Western blot bands and normalization of protein expression are as stated in Fig. 5. Values are means+s.e.m. (*hsf1*, *n*=9–10; *hsp70a* and *hsp90aa*, *n*=9–11; *hsp47*, *n*=7–9; HSP70, HSP90 and HSP47, *n*=5–6). Statistical differences between gene expression values were determined by Kruskal–Wallis one-way ANOVA followed by *post hoc* Dunn's test (*hsf1*, *hsp70a*, *hsp90aa and hsp47*, *P*<0.001). HSP90 protein expression was square-root transformed prior to analysis; statistical differences between protein expression values were determined by one-way ANOVA followed by *post hoc* Holm–Šidák tests (HSP90, *P*=0.288; HSP47, *P*=0.019) or a Kruskal–Wallis one-way ANOVA followed by *post hoc* Dunn's test (HSP70, *P*=0.002). Values for a given parameter that do not share a common letter are significantly different from one another.

In the ovary, *hsf1* expression in the cycling hypoxia treatment was ∼50% lower than in the control and combined exposure treatments (Fig. 6A; *H*=18.15, d.f.=3, *P*<0.001). In contrast, *hsp70a* mRNA levels were increased in the cycling temperature and combined exposure treatments compared with the control treatment (∼26.5- and ∼45.9-fold respectively), and were also ∼18.8-fold higher in the combined exposure treatment than in the cycling hypoxia treatment (*H*=32.35, d.f.=3, *P*<0.001). Also, relative to the control conditions, *hsp90aa* expression increased in the hypoxia (∼2.4-fold) and combined exposure treatments (∼2.2-fold; *H*=16.93, d.f.=3, *P*<0.001), and *hsp47* mRNA levels increased in the cycling temperature (∼2.7-fold) and combined exposure treatments (∼4.2-fold; *H*=20.27, d.f.=3, *P*<0.001). At the protein level, HSP70 expression was higher in the cycling temperature and combined exposure treatments compared with both the control (∼6.9- and ∼3.0-fold, respectively) and hypoxia (∼5.7-fold and ∼2.5-fold, respectively) treatments (Fig. 6B; *H*=14.34, d.f.=3, *P*=0.002). Meanwhile, ovarian HSP90 levels did not differ across treatments (*F*=1.36, d.f.=3, *P*=0.288), and there was a ∼50% reduction in HSP47 expression in the combined exposure treatment relative to the cycling hypoxia treatment (*F*=4.34, d.f.=3, *P*=0.019).

**Fig. 7.**
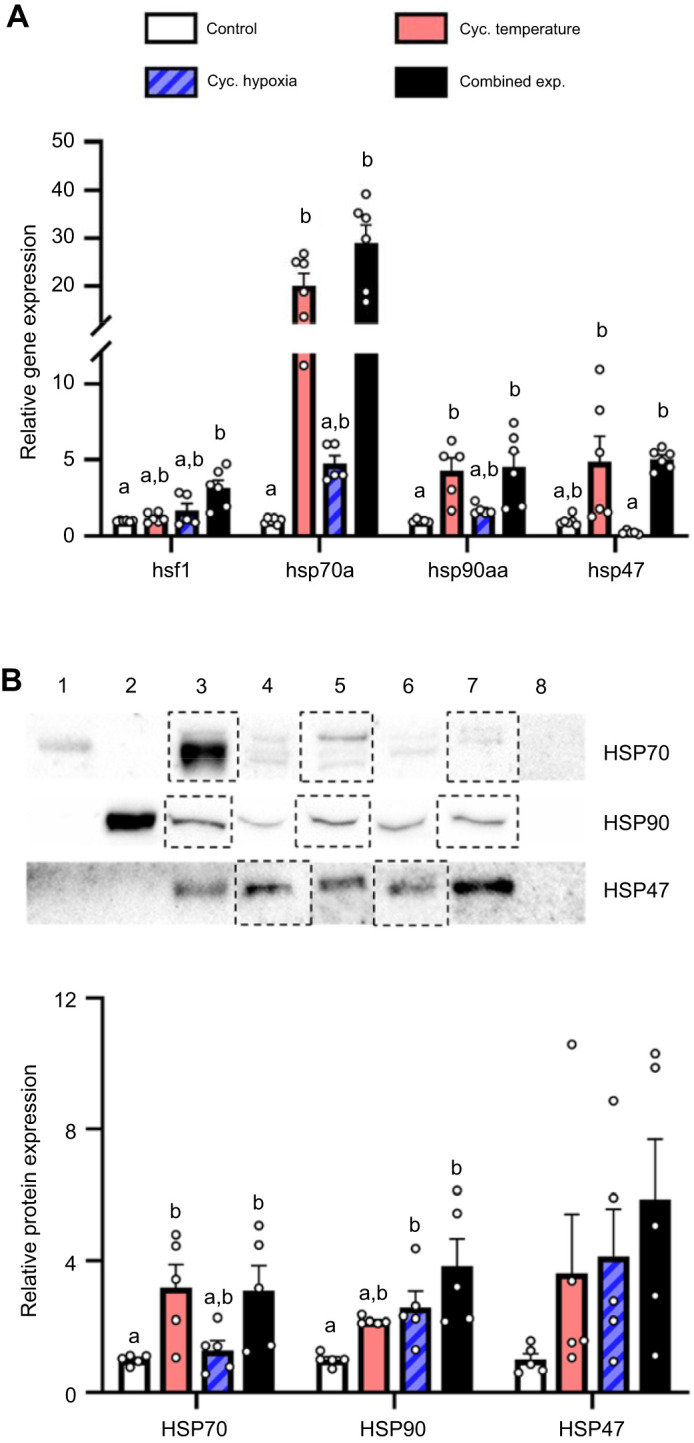
**Effects of parental treatment on zebrafish embryo cellular stress response.** (A) *hsf1*, *hsp70a*, *hsp90aa* and *hsp47* relative gene expression, and (B) representative western blot and HSP70, HSP90 and HSP47 relative protein expression in ∼1 hpf embryos derived from adult zebrafish exposed to the experimental treatments. Gene expression data were normalized and expressed as stated in Fig. 2. Western blot bands and normalization of protein expression are as stated in Fig. 5. Values are means±s.e.m. (*hsf1*, *hsp70a*, *hsp90aa and hsp47*, *n*=5–6; HSP70, HSP90 and HSP47, *n*=5). Statistical differences between gene expression values were determined by Kruskal–Wallis one-way ANOVA followed by *post hoc* Dunn's test (*hsf1*, *P*=0.014; *hsp70a*, *P*<0.001; *hsp90aa*, *P*=0.002; *hsp47*, *P*<0.001). HSP70 protein expression was log-transformed prior to analysis; statistical differences between protein expression values were determined by one-way ANOVA followed by *post hoc* Holm–Šidák tests (HSP70, *P*=0.010; HSP47, *P*=0.175) or a Kruskal–Wallis one-way ANOVA followed by *post hoc* Dunn's test (HSP90, *P*=0.004). Values for a given parameter that do not share a common letter are different from one another.

The embryos originating from females subjected to the combined exposure treatment had *hsf1* mRNA levels that were increased ∼3.1-fold relative to the control treatment (Fig. 7A; *H*=10.19, d.f.=3, *P*=0.017). Embryos in the cycling temperature and combined exposure treatment had higher *hsp70a* (∼20.0- and ∼28.9-fold, respectively) and *hsp90aa* (∼4.3- and ∼4.5-fold, respectively) mRNA levels compared with those of the control treatment, while those exposed to cycling hypoxia did not differ from any other treatments (*hsp70a*, *H*=19.02, d.f.=3, *P*<0.001; *hsp90aa*, *H*=15.18, d.f.=3, *P*=0.002). Although *hsp47* expression in the cycling temperature and combined exposure treatments were increased ∼5.0-fold relative to the control treatment and ∼21.0-fold relative to the hypoxia treatment, only the differences with the cycling hypoxia treatment reached statistical significance (*H*=17.56, d.f.=3, *P*<0.001). At the protein level, HSP70 expression was higher in the cycling temperature (∼2.9-fold) and combined exposure (∼2.8-fold) treatments compared with the control treatment (Fig. 7B; *F*=5.26, d.f.=3, *P*=0.010). In contrast, HSP90 expression was higher in the cycling hypoxia (∼2.6-fold) and combined exposure (∼3.8-fold) treatments compared with the control treatment (*H*=13.47, d.f.=3, *P*=0.004). Finally, although all three cycling treatments had HSP47 protein levels that were increased ∼3.6- to ∼5.9-fold compared with the control treatment, the response was variable and did not reach statistical significance (*F*=1.871, d.f.=3, *P*=0.175).

## DISCUSSION

Several studies have shown that environmental stressors can have lasting effects on the performance of fish across generations, but the mechanisms responsible for these responses are poorly understood (e.g. Ho and Burggren, 2012; Le Roy et al., 2017). Here, we provide original evidence that chronic exposure to environmentally relevant diel cycles of thermal stress (27–36°C) and/or hypoxia (20–85% DO) can affect parental progeny investment in cortisol and HSPs, key effectors of the endocrine and cellular stress responses, respectively. Overall, our results demonstrate that the endocrine and cellular stress responses make environmental stressor-specific and divergent contributions to non-genetic inheritance.

### Effects of cycling temperature and/or hypoxia on adult female body condition and spawning success

Relative to fish kept at 27°C and under normoxic conditions, chronic exposure to diel cycles of thermal stress, hypoxia or the combined treatment did not affect adult zebrafish body condition. Similarly, chronic exposure to diel temperature cycles above the thermal optimum in Atlantic salmon (*Salmo salar*; Morissette et al., 2021) or to fluctuating hypoxic conditions in rainbowfish (*Melanotaenia utcheensis*; Flint et al., 2018) did not significantly alter condition factor. While chronic exposure to stressful conditions or exogenous cortisol can reduce body condition in various fish species, shorter increases in cortisol associated with daily acute stressors may not be sufficient to recruit the catabolic and growth-suppressing effects of cortisol (Barton et al., 1987; McCormick et al., 1998; Madison et al., 2015; Sadoul and Vijayan, 2016). Similarly, while sustained cortisol levels and chronic exposure to species-specific thermal maxima and hypoxic conditions can reduce reproductive performance in fishes (Wu, 2009; Fuzzen et al., 2011; Pankhurst and Munday, 2011; Pankhurst, 2016; Alix et al., 2020; Servili et al., 2020), overall, the cycling thermal and hypoxic conditions used in this study did not affect fecundity or egg viability relative to the control treatment. Interestingly, however, fish exposed to the cycling temperature treatment had higher fecundity and lower egg viability than those exposed to the cycling hypoxic conditions. These results may be explained by the propensity of elevated temperatures below species-specific thresholds to simultaneously increase oocyte proliferation and reduce egg quality (Alix et al., 2020), whereas hypoxic conditions consistently reduce fecundity in proportion to the severity and duration of the challenge (Wu, 2009; Pankhurst, 2016). Reflecting the opposite effects of the elevated temperature and hypoxic conditions on fecundity, the combined exposure treatment in this study had no overall effect on egg production.

### Effects of cycling temperature and/or hypoxia on the GC stress response in adult females and their embryos

Consistent with their lack of effect on body condition, the 14 day diel cycles of elevated temperature and/or hypoxia in this study did not increase adult female zebrafish whole-body cortisol levels. While acute exposure to heat stress (Pérez-Casanova et al., 2008; LeBlanc et al., 2012; Shimomura et al., 2019) or hypoxia (Bernier and Craig, 2005; Williams et al., 2017; Mikloska et al., 2022) can elicit an increase in cortisol levels in various fish species, the impact of repeated exposure to these stressors on the endocrine stress response depends on the magnitude of the disturbance and its duration. For example, in salmonids, chronic exposure (20–35 days) to moderate diel cycles of elevated temperatures (6–8°C oscillations) had no effect on plasma cortisol levels (Thomas et al., 1986; Cassinelli and Moffitt, 2010; Chadwick and McCormick, 2017), but exposure to daily oscillations of 13.5°C for 20 days resulted in chronically elevated cortisol concentrations (Thomas et al., 1986). Similarly, in rainbow trout (*Oncorhynchus mykiss*), the pronounced increase in plasma cortisol associated with a diel hypoxia exposure (80% DO oscillation) was muted after five consecutive cycles (Williams et al., 2019). Several factors may contribute to the muted whole-body and plasma cortisol responses associated with chronic exposure to diel fluctuations in thermal and/or hypoxic conditions. For instance, chronic exposure to diel temperature cycles can increase thermal tolerance (Schaefer and Ryan, 2006; Corey et al., 2017; Cooper et al., 2021), acclimation to diel hypoxia cycles improves hypoxia tolerance (Yang et al., 2013; Borowiec et al., 2015; Williams et al., 2019), and warm acclimation can enhance the ability to cope with hypoxia (McBryan et al., 2016). Chronic stress can also modify the responsiveness of the hypothalamic-pituitary–interrenal (HPI) axis to stressors. In brook charr (*Salvelinus fontinalis*), the normalization of plasma cortisol levels associated with chronic rearing at high/stressful densities is associated with a reduction in interrenal cell adrenocorticotropic hormone responsiveness and an increase in liver cortisol metabolism (Vijayan and Leatherland, 1990). In this study, the marked treatment-induced stimulation in gill *11bhsd2* mRNA levels suggests that an increase in cortisol inactivation and excretion contributes to the regulation of whole-body cortisol levels in response to chronic diel fluctuations in thermal and/or hypoxic conditions. This conclusion is supported by observations in zebrafish that *11bhsd2* expression is up-regulated by cortisol treatment and acute stressors, that gills are a principal site of *11bhsd2* expression, and that the enzyme 11β-HSD2 plays a critical role in regulating whole-body cortisol levels (Fuzzen et al., 2010; Alderman and Vijayan, 2012; Tokarz et al., 2012, 2013; Theodoridi et al., 2021). In contrast, the lack of a treatment effect on gill *20bhsd2* expression and 20β-HC levels observed here does not support a role for the enzyme 20β-HSD2 in the muted whole-body cortisol response.

The dichotomy in whole-body and ovarian cortisol levels in response to diel cycles of elevated temperature and hypoxia highlights marked differences in cortisol metabolism between these tissues. As previously documented in adult zebrafish fed a cortisol-laced diet for 5 consecutive days (Faught et al., 2016), we observed a marked increase in ovary cortisol content without any change in whole-body cortisol levels in response to sustained diel cycles of elevated temperature and hypoxia. Although differences in cortisol compartmentalization (Faught et al., 2016) and localized follicular cortisol synthesis (Alsop et al., 2009) may contribute to the discrepancy between whole-body and ovarian cortisol content, our results also suggest differences in cortisol catabolism between these tissues. In contrast to their stimulatory effects on gill *11bhsd2* transcript levels, the diel cycles in temperature and hypoxia in this study had no effect on ovarian *11bhsd2* expression. While these results are at variance with the observation that cortisol can stimulate ovarian follicle *11bhsd2* expression *in vitro* (Tagawa et al., 2000; Li et al., 2012; Faught et al., 2016), and that 11β-HSD2 can readily convert cortisol to cortisone in ovary homogenates (Alderman and Vijayan, 2012), we suggest that the variable expression of *11bhsd2* in the ovary and the lack of an overall treatment effect observed here reflect cell type- and follicular stage-specific contributions of 11β-HSD2 to cortisol catabolism in this tissue. In the rainbow trout ovary, for example, there are marked stage-specific increases in *11bhsd2* expression during vitellogenesis and oocyte maturation, and *in situ* hybridization demonstrates a differential contribution of the theca and granulosa cells to the overall *11bhsd2* expression pattern (Kusakabe et al., 2003; Milla et al., 2006). Interestingly, the elevated ovary cortisol content in the cycling temperature and hypoxia treatment was associated with a reduction in ovarian *20bhsd2* expression, suggesting that a reduction in GC catabolism may contribute to the specific changes in cortisol metabolism observed in the ovary. Although high cortisol levels can have deleterious effects on gametogenesis in female fish, there is also compelling evidence that cortisol is necessary for oocyte maturation and ovulation (Milla et al., 2006, 2009; Faught and Vijayan, 2018; Maradonna et al., 2020; Zhang et al., 2020). Overall, we suggest that more studies are needed to understand the differential regulation of cortisol metabolism at the whole-body and ovarian levels in response to chronic stress, as well as the follicular stage-specific mechanisms involved in regulating the distinct roles of cortisol in ovarian function and non-genetic inheritance.

Our study also reports an inverse relationship between ovary and embryo cortisol content in response to diel cycles of elevated temperature and hypoxia. This observation is counter to our prediction of increased embryonic cortisol content in response to chronic heat stress and hypoxia exposure and suggests that early embryos possess a buffering capacity that prevents cortisol accumulation in response to a sustained environmental challenge. In fish, while egg cortisol levels can be increased in response to exogenous manipulations of maternal cortisol content, surprisingly few studies have explored the relationship between chronic maternal stress and embryo cortisol content (see Sopinka et al., 2017a, for a review). For example, whereas chronic physical stress of coho salmon (*O. kisutch*) during the final 2 weeks of oogenesis elevated both plasma and egg cortisol levels (Stratholt et al., 1997), neither chronic unpredictable stress in sticklebacks (*Gasterosteus aculeatus*; Giesing et al., 2011) nor chronic exposure of sockeye salmon (*O. nerka*) to a chase stressor (Sopinka et al., 2014) increased egg cortisol levels, and there was no correlation between the elevated plasma cortisol levels of migrating sockeye salmon and the cortisol content of their eggs (Taylor et al., 2016). In addition to the potential contribution of follicular somatic cells to reducing the transfer of maternal cortisol into eggs via 11β-HSD2 and GC sulphotransferase activity (Tagawa et al., 2000; Li et al., 2012; Faught et al., 2016), our observation that embryos derived from females exposed to diel cycles of elevated temperature and hypoxia have elevated *11bhsd2* and *abcb4* mRNA levels suggests that embryos also buffer against maternal cortisol transfer by increasing cortisol metabolism and excretion. However, although previous studies have shown that transcripts of various ABC transporters are maternally transferred (Long et al., 2011a,b), that efflux activity can be observed 1 h after fertilization (Fischer et al., 2013), and that blocking ABC transporters inhibits egg cortisol clearance (Paitz et al., 2016), it remains to be demonstrated that ABCB4 can transport cortisol in fish embryos. Independent of the buffering mechanism involved, given the observation that zebrafish embryo cortisol sequestration by antibody microinjection heightens the cortisol stress response in post-hatch larvae (Nesan and Vijayan, 2016), we suggest that the reduction in embryo cortisol content elicited by chronic exposure to diel cycles of elevated temperature and hypoxia could have programming effects on the HPI axis of zebrafish offspring and warrants future study.

### Effects of cycling temperature and/or hypoxia on the cellular stress response in adult females and their embryos

Diel cycles of elevated temperature and/or hypoxia elicited an HSP- and stressor-specific cellular stress response in the gills of female zebrafish. In several fish species, the mRNA levels of gill *hsp47*, *hsp70* and *hsp90a* are robust biomarkers of acute and chronic temperature stress (Healy et al., 2010; Jeffries et al., 2014; Akbarzadeh et al., 2018; Houde et al., 2019). Similarly, previous studies have shown that hypoxia exposure induces *hsp70* expression in fishes (Ton et al., 2003; Fuzzen et al., 2011; Levesque et al., 2019). In contrast, here we showed that gill mRNA levels of *hsp47*, *hsp70a* and *hsp90aa* are not biomarkers of repeated cycles of thermal stress and hypoxia exposure in zebrafish. Similarly, while the expression of several HSPs, including *hsp90*, is strongly induced by chronically elevated temperatures in annual killifish (*Austrofundulus limnaeus*), the transcript levels of these chaperones return to control levels after 2 weeks of temperature cycling, suggesting that chronic high temperatures may be more stressful than cycling conditions (Podrabsky and Somero, 2004). Moreover, in this study, while all oscillating treatments increased gill mRNA levels of the stress-inducible transcription factor *hsf1*, this response did not result in a transcriptional activation of our target HSPs. In fact, the cycling temperature and combined exposure treatments were characterized by reductions in gill *hsp90aa* and *hsp47* expression. As zebrafish possess four HSFs (Saju et al., 2018), our results question whether alternative HSFs regulate the transcription of inducible HSPs during conditions of oscillating temperature and hypoxia. At the protein level, in agreement with the findings of Chadwick and McCormick (2017) on brook trout, we observed a clear increase in gill HSP70 levels in response to diel cycles of elevated temperature. Interestingly, although cycling hypoxia by itself did not have a consistent effect on gill HSP70 levels, adult female zebrafish exposed to the combined cycling temperature and hypoxia treatment had higher gill HSP70 levels than those in the cycling temperature treatment. Given the known stimulatory effect of hypoxia on HSP70 expression in fish (Delaney and Klesius, 2004; Methling et al., 2010; Currie et al., 2010), our results suggest that by itself the cycling hypoxia exposure used in this study may not have been severe enough to induce an HSP70 cellular stress response. Similar to the previous observation in tidepool sculpins (*Oligocottus maculosus*) that heat shock increased the HSP70 response to a subsequent hypoxic exposure (Todgham et al., 2005), our results also suggest that oscillating temperatures can serve as a priming stressor in zebrafish that increases the HSP70 response to cycling hypoxic conditions. Finally, we found no evidence that cycling temperature and/or hypoxic conditions can upregulate gill HSP47 or HSP90 levels, a finding that agrees with earlier studies showing that HSP47 and HSP90 induction with heat stress in various fish tissues is smaller than the HSP70 response (Murtha and Keller, 2003; Rendell et al., 2006).

Interestingly, the oscillating temperature and/or hypoxia treatments elicited a very different cellular stress response in the ovary than in the gills at the transcript level, but a conserved HSP70 induction in response to elevated temperatures. While *hsp47*, *hsp70* and *hsp90* can have similar transcriptional responses to thermal stress across tissues (see Akbarzadeh et al., 2018, for a review), there are multiple examples of tissue-specific cellular stress responses to both elevated temperature and hypoxic conditions in fish (Dyer et al., 1991; Airaksinen et al., 1998; Delaney and Klesius, 2004; Rendell et al., 2006; Methling et al., 2010; Wang et al., 2016; Mackey et al., 2021). To our knowledge, this is the first study to characterize the effects of oscillating temperature and/or hypoxia on the expression of HSPs in the ovary. In general, our results are consistent with previous fish studies showing that ovary *hsp70* mRNA levels increase in response to an acute heat shock (Rabergh et al., 2000) or to mild chronic temperature increases (Mahmoud et al., 2020), but runs counter to decreases in ovary *hsp70* and *hsp90* expression associated with chronic exposure to high temperatures that negatively affect reproduction (Mahanty et al., 2019; Mahmoud et al., 2020). Similarly, reflecting the magnitude of the disturbance and its impact on reproduction, cycling hypoxia exposure had no effect on ovary *hsp70* transcripts in this study, but chronic exposure to hypoxic conditions that inhibit ovulation downregulate ovary *hsp70* mRNA levels in zebrafish (Martinovic et al., 2009). In this study, the primer used to quantify *hsf1* expression amplified identical regions of the two HSF1 isoforms found in zebrafish (Rabergh et al., 2000). Therefore, beyond the potential contribution of alternative HSFs (Saju et al., 2018), we suggest that the known differential regulation of *hsf1a* and *hsf1b* in the ovary and gills may have contributed to the tissue-specific effects of our treatments on *hsf1* expression (Rabergh et al., 2000). Overall, our results demonstrate that diel cycles of temperature or temperature and hypoxia induce robust increases in ovarian *hsp70a* mRNA levels and parallel, albeit variable, increases in HSP70. While these treatments also elicited more modest increases in ovary *hsp47* and *hsp90aa* transcript abundance, these transcriptional changes did not affect ovarian HSP47 and HSP90 levels.

Our study also provides the first evidence of increased embryonic HSPs following parental exposure to environmental stressors in a vertebrate species. Consistent with a previous study in European eels (*Anguilla anguilla*; Kottmann et al., 2021), we observed that the mRNA levels of all HSPs quantified in the ovary were associated with their mRNA abundance in the embryos prior to zygotic transcription. In addition, we demonstrated that parental exposure to diel cycles of elevated temperature, with or without hypoxia, results in a marked embryonic deposition *hsp70a* mRNA, smaller deposits of *hsp90aa* and *hsp47* transcripts, increases in embryonic HSP70 and HSP90 content, and elevated but highly variable HSP47 levels. In contrast, while parental exposure to diel cycles of hypoxia alone did not affect the maternal transfer of HSP transcripts, or the embryonic content of HSP70, it increased HSP90 deposits and, consistent with the effects of cycling temperature, resulted in elevated but highly variable HSP47 levels. Although the physiological consequences of these findings remain to be determined, HSPs are known to play essential roles in successful embryogenesis and maintaining cellular homeostasis in response to stressors (Rupik et al., 2011). For example, in zebrafish embryos, *hsp70* and *hsp90* are required for muscle and eye development (Lele et al., 1999; Blechinger et al., 2002; Krone et al., 2003), and *hsp47* has been linked with collagen processing events, skeletal growth and cell proliferation (Bhadra and Iovine, 2015). The fact that increased maternal loading of *hsp23* transcripts protects *Drosophila* embryos from heat-induced defects in larval performance (Lockwood et al., 2017) also suggests that the increased maternal HSP deposits elicited by diel cycles of elevated temperature and hypoxia in this study may help prepare offspring to cope with similar disturbances.

The differential impact of the cycling temperature and the cycling hypoxia treatments on the parental progeny investment of HSP mRNAs and proteins, and the fact that hypoxia overall did not increase the heat stress-induced maternal loading of HSPs in the combined exposure, are notable findings of this study. These environmental stressor-specific differences in maternal deposits of HSPs may be due in part to the specifics of our experimental design. To expose fish to environmentally realistic oscillations in oxygen levels and temperatures, the DO cycle in this study reached minimum values at the end of the dark phase of the photoperiod (05:00–08:00 h) and the temperature cycle peaked at the end of the light phase (17:00–20:00 h), i.e. 1 and 13 h prior to embryo collection and tissue sampling, respectively. Therefore, relative to when embryos and adult tissues were sampled, fish exposed to the cycling temperature treatment were given an additional 12 h to recover from the environmental stressor relative to the fish exposed to the cycling hypoxia treatment. Additionally, we suggest that the environmental stressor-specific differences in maternal deposits of HSPs may have a biological basis. For example, adult zebrafish may differ in the magnitude and speed of the acclimation effect to chronic cycles of thermal stress and hypoxia. Such differences in acclimation capacity could in return affect HSP induction and parental progeny investment. Alternatively, despite the fact adult zebrafish were exposed to 20% DO for 3 h daily, conditions which approach the critical O_2_ tension (*P*_crit_) in this species (Mandic et al., 2020), it is possible that the hypoxic conditions used in this study caused less protein to denature than the thermal regime and therefore induced a smaller cellular stress response.

Our study focused on the proposed maternal contribution of signals into their embryos, but paternal contributions can also affect offspring phenotype. For example, epigenetic signatures in sperm (e.g. miRNA levels, DNA methylation) can alter offspring stress responses and behaviour (Rodgers et al., 2015; Ord et al., 2020). Indeed, in zebrafish, maternal methylation patterns may be discarded early in development, and offspring develop similar DNA methylation patterns to their fathers (Jiang et al., 2013), and these DNA methylation patterns can cause transgenerational effects through the paternal line (Valdivieso et al., 2020). As such, both maternal and paternal signal transfer likely determine offspring phenotypes.

In conclusion, this study provides original evidence that parental progeny investment of cortisol and HSPs is dynamic and responsive to environmental conditions. Specifically, we demonstrated that parental exposure to chronic diel cycles of thermal stress and/or hypoxia can affect the embryonic deposition of cortisol and inducible HSPs, key effectors of the endocrine and cellular stress responses known to play various regulatory roles during embryonic development. Whether these transgenerational signals of environmental stress help shape offspring phenotype and contribute to climate change resilience as predicted by the maternal match hypothesis is the subject of ongoing studies.

## Supplementary Material

10.1242/jexbio.244715_sup1 Supplementary information
