# Supplementary material for: Zebrafish parental progeny investment in response to cycling thermal stress and hypoxia: deposition of heat shock proteins but not cortisol
Source: J Exp Biol. 2022 Nov 11;225(21):jeb244715. doi: 10.1242/jeb.244715 (PMC9687554; doi:10.1242/jeb.244715)
Supplement: Supplementary information [file jexbio-225-244715-s1.pdf]

Table S1. Compound-specific LC-MS/MS parameters for the steroid analytes investigated in this study. Parameters include retention time, parent (Q1) and daughter (Q3) ion fragments, and the corresponding limits of detection for analytes (cortisol, cortisone, 20β–hydroxycortisone) and standards (cortisol-d4, 17β-estradiol 3-benzoate).

Compound	Retention time (min)	Q1 fragment (amu)	Q3 fragment (amu)	Limit of detection (pg ml^{-1})
Cortisol	6.47	361.1	91.0	800
Cortisone	6.63	363.1	327.1	400
20β–hydroxycortisone	6.37	363.1	163.2	400
cortisol-d_4	9.53	377.1	105.1	200
17β-estradiol 3-benzoate	6.63	367.2	121.0	400

Journal of Experimental Biology • Supplementary information

Table S2. Nucleotide sequences of zebrafish primers used for quantitative real-time PCR.

Gene	Accession no.	Sequence (5' – 3')	Efficiency (%)
11bhsd2	NM_212720.2	F: TGCTGCTGGCTGTACTTCAC<br>R: TGCATCCAACTTCTTTGCTG	92.7
20bhsd2	KM_279631.1	F: GCTGGGCTGTTGTAACTGGT<br>R: TTGGCAAGCTCTTCAGCATA	107.3
abcb4	NM_001316714.1	F: CTGGCAGGACATGCTCTAAA<br>R: TTCTCAATGGCTTCTGTTGC	94.3
ef1α	NM_131263.1	F: GGGCAAGGGCTCCTTCAA<br>R: CGCTCGGCCTTCAGTTTG	102.2
hsf1	NM_001313736	F: ATCACATTAGCACGGTCCAA<br>R: GAGCTGCTGTCGTTCAACAT	96.9
hsp47	NM_131204.2	F: CAATGTCTTCCATGCCTCCT<br>R: AGCTTGGGGTTCCTCATCTT	97.1
hsp70a	AF_210640	F: AAGATCACCATCACCAACGA<br>R: TGCACCATTCTCTCGATCTC	102.4
hsp90aa	NM_131328	F: CGGTTACCCAATTACGCTTT<br>R: TTTCTCGCCTTCCTCAAGAT	102
rpl13a	NM_212784.1	F: ATGCTTCCACACAAAACCAA<br>R: CATGCGCTTTCTCTTGTCAT	101.1

abc, ATP binding cassette; *bhsd,* beta hydroxysteroid dehydrogenase; *ef1α,* elongation factor 1*α*; F, forward; *hsf,* heat shock factor; *hsp,* heat shock protein; R, reverse; *rpl13a*, ribosomal protein L13A.

Journal of Experimental Biology • Supplementary information
